# Stem cell preservation for regenerative therapies: ethical and governance considerations for the health care sector

**DOI:** 10.1038/s41536-020-00108-w

**Published:** 2020-12-01

**Authors:** Zubin Master, Aidan P. Crowley, Cambray Smith, Dennis Wigle, Andre Terzic, Richard R. Sharp

**Affiliations:** 1 Biomedical Ethics Research Program, Mayo Clinic, Rochester, MN 55905 USA; 2 Center for Regenerative Medicine, Mayo Clinic, Rochester, MN 55905 USA; 3 College of Science, Department of Biological Sciences, University of Notre Dame, Notre Dame, IN 46556 USA; 4 School of Medicine, University of North Carolina-Chapel Hill, Chapel Hill, NC 27599 USA; 5 Department of Surgery, Mayo Clinic, Rochester, MN 55905 USA

**Keywords:** Medical ethics, Regeneration

## Abstract

The stem cell preservation industry has grown substantially with private businesses, public hospitals, and academic medical centers considering preserving induced pluripotent stem cells, mesenchymal stem cells, and other cell types of patients and the public in order to potentially use them for stem cell therapy should such an intervention exist in the future. Despite this growth and interest among private firms and academic centers, no study has yet considered the bioethical issues of such platforms. In this article, we explore several ethical and social issues related to the biopreservation of stem cells for future regenerative therapies. We analyze a range of bioethical considerations that public and private institutions should bear in mind as they develop stem cell preservation platforms. These include medical validation of regenerative interventions and their influence on the public understanding of stem cell therapies, the impact of public trust of organizations creating a private, for-profit venture of stem cell preservation, and logistical issues in the governance of the collection including ownership and dispositional authority, informed consent and access, and withdrawal and non-payment. These considerations should be incorporated into current and future stem cell preservation platforms in order to promote the responsible translation of regenerative medicine.

## Introduction

Public interest in stem cell therapies continues to grow as advances in regenerative science and clinical applications progress^1–3^. Current cell-based therapies, for example, include treatment for cancers of the blood or immune system, such as lymphoma or leukemia^4^, as well as investigational uses for heart failure, neurologic disorders, musculoskeletal indications, and autoimmune diseases^5,6^. The Alliance for Regenerative Medicine’s latest report shows a $2.6 billion total global financing for cell and gene therapies, with over 959 companies, and 1052 clinical trials underway, most of which are in phase 2^7^. Science driven early successes are poised to transform the regenerative horizon, further fueled by the expansion of the regenerative toolkit^8,9^.

Focusing here on the potential of stem cell-based therapies, there is general interest in preserving healthy young stem cells, collected and stored for future use^10^ as stem cells have been shown to decrease in their ability to proliferate as they age^11,12^. Ultimately, the aim of stem cell preservation is that should one fall ill and require the use of stem cells, and should future regenerative medicine treatment options exist, one’s own stem cells will be available for therapeutic use. Stated more succinctly, the goal is to bank healthy stem and possibly other cell types as a form of bioinsurance should they be needed later in life.

Stem cell preservation for future medical treatment via public and private umbilical cord and tissue banks have existed for over 20 years^13–15^. However, biospecimen banks are now expanding to offer private banking of adult cells, including mesenchymal stem cells (MSCs) and induced pluripotent stem cells (iPSCs), as well as banking T-cells for CAR-T therapy^16,17^.

Adult stem cell banking is attracting interest due to a constellation of factors, including the enthusiasm surrounding stem cells and regenerative medicine, national and international initiatives to stockpile stem cells, and the presence of an existing cord blood market suggesting a viable business model^18–21^. Cord blood is collected from the placenta or umbilical cord at birth and can be stored in public or private banks. In fact, there are estimates of 800,000 units of cord blood stored in public banks and 5,000,000 units stored in private banks^22,23^. Derived hematopoietic stem cells (HSCs) can be used in principle to treat over 80 disorders including blood cancers, blood disorders, metabolic disorders, and immune system disorders^24^. The first cord blood transplant was conducted in 1988 and to date, over 40,000 transplants have been performed^25^. And for the most part, the Canadian and U.S. media on umbilical cord blood portray therapies in alignment with clinical evidence^26^. Although there are many approved treatments using HSCs, there are currently no Food and Drug Administration (FDA) approved treatments using iPSCs or MSCs, the latter being typically derived from adult adipose or other tissue including dental pulp. In about 2026, the global cord blood banking market is estimated to reach over $20 billion US^27^ and the adult stem cell banking market is estimated to reach over $13 billion US^28^.

Although a validated cell preservation or banking model is contingent on regenerative medicine therapies becoming standard-of-care and on younger stem cells being more viable or superior for transplantation than older ones, companies have begun to market stem cell preservation services. Currently in the United States, several private companies advertise services for the isolation and storage of different biomaterials including cord blood and tissue, peripheral blood, adipose tissue, dental pulp, and placental tissue. These companies report on the current uses of HSCs, and the future potential uses of MSCs and iPSCs for a range of treatments for diseases including heart disease, spinal cord injury, and diabetes among others.

Several of the ethical, legal, and social (ELS) issues about the cord blood banking apply to the stem cell preservation market which uses MSCs and iPSCs. Among them, topics such as ownership and disposition authority, disclosure of information during informed consent, and withdrawal and non-payment are particularly relevant to stem cell preservation^14,26,29–35^. In addition, new ELS issues also deserve consideration about private stem cell preservation, including medical validation and trust. We analyze these particular ethical and social issues in the context of stem cell preservation to inform health care institutions considering such an endeavor because they hold moral obligations to the public they serve and as health care providers, they may be perceived as having dual, and possibly conflicting interests. Private businesses also need to consider ELS issues for similar reasons of accountability and responsibility. In particular, we discuss how stem cell preservation may inadvertently medically validate the utility of stem cells for future regenerative therapies, how public perceptions of trust can impact an organization’s commitment to uphold a central mission to serve the interests of patients, and how issues of ownership and disposition, informed consent, and withdrawal and non-payment are implicated in governing the collection. An understanding of the ELS issues would serve to help organizations ensure transparency and accountability, ethical communication to the public, and good governance over the biosamples.

## Medical validation and the public’s understanding of stem cell preservation

A major concern in offering stem cell preservation services is whether doing so effectively implies that cell banking meets a critical future need, implying an indirect endorsement of advances in regenerative medicine. This issue is most relevant to academic medical centers that are considered by the public to be trustworthy stewards of medical innovation and in the development of treatments for public benefit. While prudence about storing cells now for future therapies forms the basis of this practice, offering a private banking service to preserve stem cells may suggest the notion that storing cells will enable future therapeutic use, which assumes much about the advancement of the field and the future needs of patients. To avoid reinforcing these and other perceptions that may not (yet) be fully grounded in scientific evidence, organizations should consider developing accurate and responsible information access and educational practices aimed to better appraise the public.

As with any service, properly informing consumers through a robust information-sharing platform is key to preventing misunderstanding of the services being offered. This is particularly essential for this highly vulnerable population of patients and their families which are in need of a regenerative therapy most of whom have exhausted conventional options^36^. As future application of stored cells is by no means guaranteed because it is contingent on the development of treatments, organizations should emphasize that users are paying solely for stem cell isolation and storage services and that there is no guarantee a client will need these cells or that a treatment will exist where the cells could be used in the future. It is important to reinforce this message in various contexts when sharing information, including on public websites, pamphlets and brochures, in-person discussions, and in written contracts. Repetition is critical to counter the aggressive promotion seen in the yet-unapproved marketing of stem cell therapies^37–40^, which is likely to distort public understanding of the current state of stem cell science and may make it difficult for the stem cell preservation bioindustry to explain that there are no assurances to such an investment.

In a similar vein, advertising around stem cell preservation services must be rigorous and factual. Advertisements that tout messages such as *an investment now could save your life later* or *you owe it to yourself and your family* should be avoided as such messaging suggests that an investment is necessary and may even elicit feelings of guilt for not investing in banking one’s cells. While these are not actual advertisements, several studies suggest that private umbilical cord blood and stem cell banks may be over promoting the benefits of such biorepositories^14,26,41^. When examining websites of private cord blood and stem cell banks in Canada, researchers found a high use of emotionally-charged testimonials portraying successful patient stories and excitement about the future of regenerative and stem cell science, skewed numerical estimates of potential future uses of stored cells, and advertisements centered on peace-of-mind and responsible decision-making^41^. An analysis of North American news portrayals suggests an overall negative perception of private cord blood banks compared to public banks because of their use of strong narratives and promotion of benefits whereas public cord blood banks are seen to advertise more in alignment with clinical evidence^26^. As studies of parents interested in cord blood banking show low levels of knowledge^42–46^, particularly in the uses of cord blood^44,47^, we might expect similar levels of deficits in knowledge among people interested in stem cell preservation. The use of advertisement and promotional material that may be misrepresented by stem cell preservation organizations is likely to impact a potential client’s ability to fully understand and consent to biopreservation services as we have seen in the cord blood banking space.

Along with advertising and messaging, the name of the stem cell preservation organization itself can be highly suggestive. For example, calling stem cell preservation ventures “BioInsurance” can easily carry with it the same connotations of the term “insurance.” This may imply that storing your cells will definitely protect or insure you against a possible future in which these cells are needed for treatment. The language of “insurance” connotes paying for certain coverage for unforeseen circumstances, whereas stem cell preservation does *not* guarantee future protection or coverage in the event of illness. More accurate names for these stem cell preservation banks might be StemVault, CellStore, or StemPreserve, which suggests that they store cells, but do not guarantee they will be used for future health care needs.

It is thus important that promotional, educational, and advertisement materials accurately portray stem cell preservation services in order to avoid misperceptions or misinterpretation by the public.

## Perceptions of trust for private biorepositories

Cord blood banks can use donation systems that are publicly available to others, for private use, or a hybrid model. Public banks are usually non-profit, found in hospitals, universities, and charitable organizations, have no collection or storage fees, and donations are available to the public for allogenic transplantation but can also be used autologously. Private or commercial banks charge for collection and storage, and focus on autologous use of the donor or by another person, i.e., a family member. The boundaries of such models are fluid as a hybrid system can be used to maintain a private donation that can also be used publicly. Current stem cell biopreservation banks that store adult stem cells seem to be commercial banks but some permit both private and public storage^41,48^. In the future, academic medical centers may consider creating adult stem cell preservation platforms if regenerative medicine applications become more common. To this, a highly educated workforce proficient in regenerative medicine will be needed^49^. There is a substantive amount of evidence suggesting that a commercialization ethos can impact the level of trust in biorepositories^50,51^. Studies of different stakeholder groups have found that trust in university-funded scientists is high and declines when scientists work for private, for-profit companies^52–55^. The issue of public trust is especially important for academic medical centers that serve a role in providing health care services to the community. As academic medical centers consider establishing private stem cell preservation services, they will need to consider the impact of that activity on public perceptions, including the possibility that contracting with private preservation companies could result in a loss of trust in the academic organization’s commitment to their central mission of public service.

There are several reasons that help to explain why the public perceives privatization and researchers within it as more self-interested than interested in the public good. Private institutions are considered to be profit-driven, sometimes at the expense of consumers^50^. Consumers who provide samples to a public biorepository may feel that they have less control over how their samples are used or whether those samples might be shared with other organizations that they view as less trustworthy. A private stem cell preservation company may be perceived as unfairly catering to the wealthy and not to other members of the public who cannot afford such services, thereby reinforcing a perception that regenerative therapies will only be available to the affluent. These and other opinions may decrease public trust in medical institutions whose main mission is to meet the health care needs of their patients and the communities they serve.

Public skepticism about biobanking companies with commercial motives might suggest that medical institutions are better poised to offer future stem cell preservation services than their private counterparts. Given the heightened trust in publicly funded scientific institutions, a medical center offering cell preservation services may actually be seen as more trustworthy or regulated. Because of their other laudable pursuits, publicly funded institutions may be viewed as more interested in the public good. Well-established medical institutions’ familiarity with biologically relevant regulatory and legal structures, and trained regenerative specialists^56^, may protect patient interests better than other players developing clinical-grade cells and permitting ethically questionable disposition options. Members of the public may also feel that academic medical centers are less likely to exaggerate the potential benefits of stem cell banking or promulgate false information regarding the future of regenerative medicine.

Medical institutions interested in the development of a stem cell preservation service should consider forms of patient and community engagement to determine whether such a venture would be accepted by their patients and the public or whether it could harm the institution’s reputation. Such a study may also yield useful information in determining how best to govern these stem cell collections, whether to create a fully private or a hybrid private/public stem cell bank, and costs associated with isolation and storage. In a hybrid model, the user could partially pay for isolation and storage with the caveat that some of their biosample could be used for research or the clinical treatment of another person. Such a model may off set negative perceptions of commercial biobanks. In addition, academic medical centers or private cell preservation companies may consider investing part of their proceeds in the advancement of stem cell or regenerative medicine research. Several research studies on corporate social responsibility suggest that ethical and philanthropic investments not only serve the public good^57^, they may increase trust in companies and brand recognition which can subsequently influence consumer behavior resulting in profits^58–60^. Using profits from stem cell preservation services for socially laudable goals such as advancing regenerative medicine science or promoting public education in regenerative medicine would support the academic medical center’s broader mission and potentially offset possible negative perceptions associated with privatization and commercial banking. Similar calls to enhance corporate social responsibility have been discussed to offset risks related to biomanufacturers involved in the opioid epidemic and the sales of unproven stem cell interventions^61–63^.

## Policies and governance of the collection: considerations for stem cell preservation organizations

Organizations offering adult stem cell storage services should also consider policies regarding ownership and disposition of stem cells, as well as issues surrounding informed consent and donor capacity, non-payment, and the return of results in situations where samples may also be used for research.

### Ownership and disposition

In the context of biobanking for research purposes, many people report a perception that they are the owners of their donated biological samples and health information^52,54^. This perception may reflect a desire to have some level of control over the disposition of their donated biomaterials. In the context of research-related biobanking, samples are owned by institutions, but donor information and sample-sharing practices depend on the consent model adopted by the biobank. While many research biobank participants are open to sharing their samples for what they feel are morally acceptable goals in understanding disease and the development of novel diagnostic and treatment interventions, multiple studies have shown that donors wish to have greater control over uses of their biospecimens in ethically contentious research (e.g., research on genes that contribute to alcoholism or schizophrenia) and greater control over the use of their samples by organizations they view as less trustworthy, such as law enforcement agencies or insurance companies^54,64–66^.

Organizations developing stem cell preservation services need to consider issues of ownership and disposition, which depend on whether the cell bank is fully private or private/public hybrid. A fully private stem cell preservation model in which clients pay for the isolation and storage of cells may imply that clients have full dispositional authority over how their cells are used. However, there may be significant concern should a client want to use their cells in the pursuit of a risky or unproven regenerative medicine therapy. If an unproven treatment using privately stored stem cells harms the patient, there could be concerns of liability for the storage company. Stem cell preservation companies, therefore, need to consider whether clients would be allowed to use their cells for any future purpose or whether those future uses would be subject to some level of oversight and approval by the bank. This requires stem cell preservation organizations to develop policies on whether full control is in the hands of the client or whether cells can only be used to pursue regulatory approved treatments or experimentally valid interventions such as clinical trials. Clients must be made aware of the bank’s policies on these and other related matters during the consent process. While organizations could require that cells only be used for validated treatments, questions about international use become relevant, since patients could take their cells to countries where there are differences in regulations or there is less oversight for cellular therapies; case in point, one private adult stem cell bank sends their clients to receive MSC therapy in a hospital outside the United States^67^. Many clinics offering unproven stem cell interventions advertise them on clinical registries as pay-to-participate clinical trials^68^, but there is often only limited effort by such companies to systematically collect data or to design a controlled trial. To develop clear and broadly applicable policies on valid treatments and experimental interventions is difficult, but these are important policy considerations for institutions offering adult stem cell preservation services.

Clients may also wish to use iPSCs to create gametes to be used for reproductive purposes. Many scholars have discussed the ethics of using pluripotent stem cells for reproduction^69–73^. One primary concern here lies in the safety of such experimental interventions on the resulting child. In addition to reproductive purposes, clients may also wish to use their cells for maintaining youth or for self-treatment. Relatively recently, fitness enthusiast and Do-It-Yourself (DIY) biohacker Ben Greenfield injected himself with MSCs obtained and stored from him when he was younger in order to maintain his youth, physical fitness, and vitality^74^. Gene editing technologies have also been used by DIY supporters to treat HIV or lactose intolerance, and build muscles among other purposes and gene editing technologies can be applied within stem cells used for transplantation^75^. The main concern here is that DIY stem cell treatments pose substantial safety concerns. In addition to self-use, clients may consider donating stem cells or using them for morally questionable research. Given that it is unclear what clients may use their stored stem cells for, stem cell preservation services need to develop rigorous policies and reliable approaches to determine the extent of allowable uses of stored stem cells. For example, they may consider a conservative policy that allows the use of cells only for personal treatment and prohibit the use for third-party research or reproductive purposes. However, limiting the use of privately stored stem cells may be considered an infringement on the client’s perception of ownership of their biomaterials and their desire to do whatever they want with their stored cells. Stem cell preservation services may alternatively adopt an approach in which a diverse committee of experts and community members, both within and external to the company, review each requested use of stored cells on a case-by-case basis to render a decision. Cell preservation companies may also require third-party oversight, such as by an Institutional Review Board, prior to using a client’s stem cells for research purposes. The use of third-party oversight bodies in research biobanks has been suggested as a way to preserve the trust of donors when using their samples for future research^76–78^. Such governance models may limit the desire for control by potential clients and may offer a way to balance the best interests and protection of clients, the biopreservation organization, and society.

### Informed consent and access

Informed consent is a cornerstone of research ethics^79^. Depending on the model for stem cell preservation, an organization should consider several important aspects of informed consent. In the context of stem cell preservation services, obtaining informed consent for the isolation and storage of adult stem cells is critical to ensure that clients fully understand the collection and storing processes, the risks, the obligations of the organization, and what is expected of clients and the organization. Policies surrounding access, disposition, withdrawal, and non-payment would need to be clearly conveyed to clients as part of obtaining consent and signing a contract. Among these considerations, the informed consent process should reinforce the central premise that storing adult stem cells does not guarantee their use in future treatments. Stem cell preservation companies can convey this information in a variety of ways, including having discussions with clients; incorporating decision aids into the process to better outline risks, benefits, and values; and using simple and straightforward language while limiting the use of legal and scientific terms, and lengthy informed consent documents^80–83^. Stem cell preservation services should also be aware of and develop policies that address assent of minors and the transfer of ownership, control, and dispositional authority when the child becomes a legal adult. Moreover, policies surrounding surrogate guardianship and stewardship of the cells and transfer of control should a patient permanently lose capacity should be developed. A final consideration for organizations offering both public and private services is explaining to clients that their sample may not be available for their child or family member if they choose a public option, which can be used for allogenic transplantation of another person if matched. This is a feature most public, private, and hybrid cord blood banks explain when presenting options online and should be explained during informed consent^14,41^. Similar explanations might be considered important for hybrid stem cell preservation services permitting third party or public donation.

As most parents receive information about cord blood banking from print and electronic media, including internet-based advertisements and from private firms^84^, it is important to provide prospective clients considering stem cell preservation additional sources of trusted information such as from the International Society for Stem Cell Research^25^, BeTheMatch^85^, Parent’s Guide to Cord Blood^86^, and EuroStemCell^87^. By adding additional outside references, the organization would demonstrate their responsibility in ensuring that clients are well-informed prior to making a decision.

### Withdrawal and non-payment

Another question that must be considered is what would happen to a client’s stored samples should they wish to withdraw from the stem cell preservation service or have their sample destroyed. In the context of research biobanking, it is well recognized that research participants storing their samples and data have the right to withdraw for any reason and their samples and health information are destroyed^54^. However, because samples in a private stem cell preservation company are stored for personal use, policies must outline whether in cases of withdrawal, stored stem cells would be relinquished to the client or destroyed. Destruction of samples may better mitigate associated risks of withdrawal, but this condition would need to be clearly explained in the informed consent process.

Another consideration involves what would happen to privately stored cells should the client die. Would ownership be officially transferred to a family member for their use or use by others, or would the samples be destroyed? Transfer of authority could be decided on a client-to-client basis prior to storage. However, if it is not possible to contact a family member or if a family member does not want the cells, then the policy could stipulate destruction or release to a public bank for research purposes.

In addition, provisions would need to be developed for failure to pay maintenance fees or the transfer of services to other clients. Due to high upfront processing costs paid by the consumer, failure to pay small annual fees may not justify immediate destruction of samples. One company’s solution to non-payment is a 60-day grace period. If the company does not receive payment or hear from the client by that point, then the client’s cells become property of the company for research purposes^88^. Policies should also be crafted outlining the circumstances for when and what purposes storage services can be transferred to other clients.

## Conclusion

Beyond the rigor of science and regulatory compliance, the health care sector interested in biopreservation needs to consider pertinent ELS dimensions in conjunction with practical issues related to stem cell preservation. The future of regenerative medicine holds significant promise, but given the hype surrounding the health benefits of regenerative services^26,89^, and the creation of an industry of clinics marketing unproven stem cell and regenerative therapies, organizations interested in cell preservation need to manage public expectations on the future prospects of biopreservation. This is of particular relevance to health care organizations charged with the delivery of health care services as a public good. The potential of effective or perceived medical validation of a service responsive to an unpredictable future raises important issues. Organizations would do well to temper enthusiasm in marketing, have education that best informs patients, and design transparent policies on ownership and disposition, withdrawal, and issues of non-payment. The ways in which health care organizations and private companies manage these ethical and practical challenges will have a significant impact on public trust, not just in regenerative medicine, but on the broader aims of these organizations themselves. Organizations need to consider how they will position themselves in relation to what continues to be a developing area of regenerative medicine research and therapy. Further research into public, patient, and other stakeholder perceptions of private cell preservation is needed to understand the values, needs, and desires of clients and others interested in cell preservation. Such research will also help elucidate the potential role of scientific and medical societies, government agencies (i.e., the Federal Trade Commission), and accreditation bodies (i.e., the Foundation for the Accreditation of Cellular Therapy) in overseeing stem cell preservation services. Having considered issues across the ELS spectrum related to stem cell preservation will help advance the responsible translation of regenerative medicine while preserving public trust in companies providing stem cell preservation services.
